# Foot characteristics of the daily-life gait in postmenopausal females with distal radius fractures: a cross-sectional study

**DOI:** 10.1186/s12891-023-06845-5

**Published:** 2023-09-05

**Authors:** Akiko Yamamoto, Koji Fujita, Eriku Yamada, Takuya Ibara, Fumiyuki Nihey, Takuma Inai, Kazuya Tsukamoto, Yoshiyuki Kobayashi, Kentaro Nakahara, Atsushi Okawa

**Affiliations:** 1 Department of Orthopaedic and Spinal Surgery, Graduate School of Medical and Dental Sciences, Tokyo Medical and Dental University, 1-5-45, Yushima, Bunkyo-Ku, Tokyo, 113-8519 Japan; 2 Department of Functional Joint Anatomy, Graduate School of Medical and Dental Sciences, Tokyo Medical and Dental University, 1-5-45, Yushima, Bunkyo-Ku, Tokyo, 113-8519 Japan; 3 Biometrics Research Laboratories, NEC Corporation, 1131, Hinode, Abiko-City, Chiba, 270-1198 Japan; 4 QOL and Materials Research Group, Department of Life Science and Technology, National Institute of Advanced Industrial Science and Technology, Health and Medical Research Institute, 2217-14 Hayashi-Cho, Takamatsu-City, Kagawa 761-0301 Japan; 5 Human Augmentation Research Center, National Institute of Advanced Industrial Science and Technology, 2-8-5 Aomi, Koto-Ku, Tokyo, 135-0064 Japan

**Keywords:** Distal radius fracture, Accidental falls, Gait analysis, Daily life

## Abstract

**Background:**

Gait decline in older adults is related to falling risk, some of which contribute to injurious falls requiring medical attention or restriction of activity of daily living. Among injurious falls, distal radius fracture (DRF) is a common initial fragility fracture associated with the subsequent fracture risk in postmenopausal females. The recent invention of an inertial measurement unit (IMU) facilitates the assessment of free-living gait; however, little is known about the daily gait characteristics related to the risk of subsequent fractures. We hypothesized that females with DRF might have early changes in foot kinematics in daily gait. The aim of this study was to evaluate the daily-life gait characteristics related to the risk of falls and fracture.

**Methods:**

In this cross-sectional study, we recruited 27 postmenopausal females with DRF as their first fragility fracture and 28 age-matched females without a history of fragility fractures. The participants underwent daily gait assessments for several weeks using in-shoe IMU sensors. Eight gait parameters and each coefficient of variance were calculated. Some physical tests, such as hand grip strength and Timed Up and Go tests, were performed to check the baseline functional ability.

**Results:**

The fracture group showed lower foot angles of dorsiflexion and plantarflexion in the swing phase. The receiver operating characteristic curve analyses revealed that a total foot movement angle (TFMA) < 99.0 degrees was the risk of subsequent fracture.

**Conclusions:**

We extracted the daily-life gait characteristics of patients with DRF using in-shoe IMU sensors. A lower foot angle in the swing phase, TFMA, may be associated with the risk of subsequent fractures, which may be effective in evaluating future fracture risk. Further studies to predict and prevent subsequent fractures from daily-life gait are warranted.

## Background

Falls are common in older adults, which can lead to major healthcare problems such as fracture, functional decline, and hospitalization [1]. Approximately 10% of falls result in fractures[2], and distal radius fracture (DRF) is one of the most common sites of initial fragility fractures caused by falls from post-menopause to the early 70 s [1, 3]. Moreover, the initial DRF increases the risk of subsequent fracture, and the hazard ratio is approximately 6 times higher in females in their 50 s [4]. The experience of DRF has been identified as a predictor of future fracture of hip and spine [5], while there are also reports suggesting that it is not an independent predictor of worsened quality of life[6]. Much is still unknown about its relationship with subsequent fractures.

More than half of DRFs are caused by a fall, which often occurs while walking; therefore, several studies have indicated that changes in gait patterns are related to falls [7, 8]. Quantitative gait parameters such as slower gait speed, shorter stride length [9], and foot kinematics such as lower peak dorsiflexion angle of the foot [10, 11], as well as larger variability of these parameters [12], have been reported to be associated with fall risk [13, 14]. However, the gait assessments in previous studies were mainly measured for only a few minutes with specific devices in the laboratory, which may not accurately reflect daily free-living assessments because of the participants’ concentrated efforts [15].

Many wearable-based data methods have recently been investigated to measure free-living mobility behavior [16–18]. Among the numerous wearable sensors, inertial measurement units (IMU) are widely used to assess gait in free-living conditions because of their low cost, accuracy, and small size [19, 20]. Gait data obtained in daily life are useful for estimating fall risk [8, 16, 21]; however, few studies have focused on foot angle and trajectories in daily life or on the risk of subsequent fractures.

We hypothesized that the IMU in insoles may be effective in evaluating free-living gait with a precise foot trajectory. We investigated patients with DRF resulting from falls for early detection of the potential risk factors related to subsequent falls and fractures. We aimed to evaluate the foot kinematic characteristics of patients with DRF in daily gait and calculate fracture risk cutoff values.

## Methods

### Participants

In this study, we included only postmenopausal females because of the sex difference in gait characteristics and the much higher risk of falls and fractures in this group. We recruited patients with DRF who had undergone surgery for their first fragility fracture at five general hospitals. We defined these patients as the fracture group and compared their results with those of the healthy volunteers. Age-matched participants without a history of fragility fractures were recruited as the control group through local media advertisements. The inclusion criteria for this study were the ability to walk without any support, no history of lower-extremity injury, no known neuromuscular disorders, neurophysiological, or neurovascular problems that may affect gait. Fragility fractures were defined as those following a fall from standing height or less. We excluded patients with hormone replacement therapy, with DRF due to traffic or industrial accidents or multi-organ injuries, and patients with any kind of postoperative course problems, such as infection and complex regional pain syndrome.

This multicenter study was conducted in accordance with the Declaration of Helsinki guidelines. Written informed consent was obtained from all participants before participation. This study was approved by the Institutional Review Board of Tokyo Medical and Dental University (M2020-365).

### Daily-life gait assessments

**Fig. 1:**
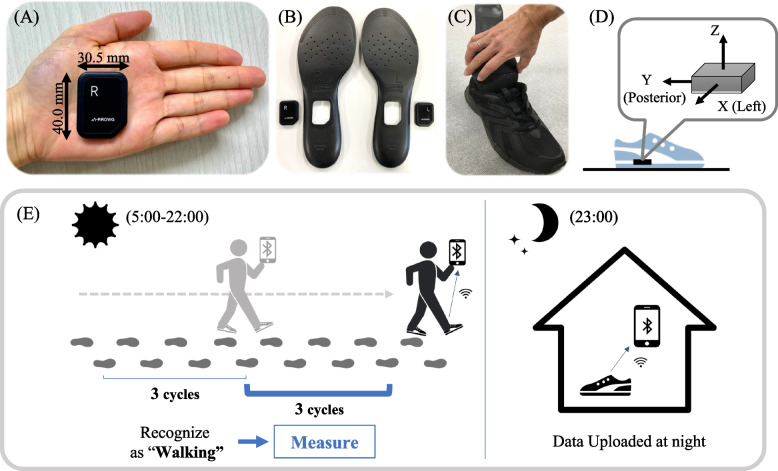
Configuration of the IMU sensors: (**A**) The IMU with the accelerometer and gyroscope; (**B**) The dedicated insoles; (**C**) The IMU in insoles were set in the participants’ own shoes; (**D**) Illustration of the orientation of the IMU relative to the global frame of the measurement; (**E**) The mechanism of measuring daily-life gait with the IMU sensors

Daily gait data were measured using in-shoe IMU sensors (A-RROWG, NEC Corporation, Japan). This IMU sensor is a small (40.0 × 30.5 × 7 mm) and lightweight (11 g) in-shoe sensor and includes a three-axis accelerometer and gyroscope (Fig. 1A). The IMU sensor in the dedicated insole was placed at the arch of the foot (Fig. 1B, C), and the X-, Y-, and Z-axes of the IMU were set along the medial–lateral, anterior–posterior, and vertical directions, respectively (Fig. 1D). When a person with these sensors walks a stable straight line over three gait cycles between 5 am and 10 pm, the in-shoe IMU sensor recognizes that the person is walking based on the acceleration in the anterior–posterior direction and saves the IMU signals of the next three gait cycles as one gait measurement [22]. The IMU signals were sampled at a rate of 100 Hz and wirelessly transferred the obtained data and time to a smartphone via Bluetooth if the participants had it with them. If a person does not have a smartphone, the data are uploaded automatically via Bluetooth at 11 pm while keeping the smartphone near the IMU sensors (Fig. 1E).

**Fig. 2:**
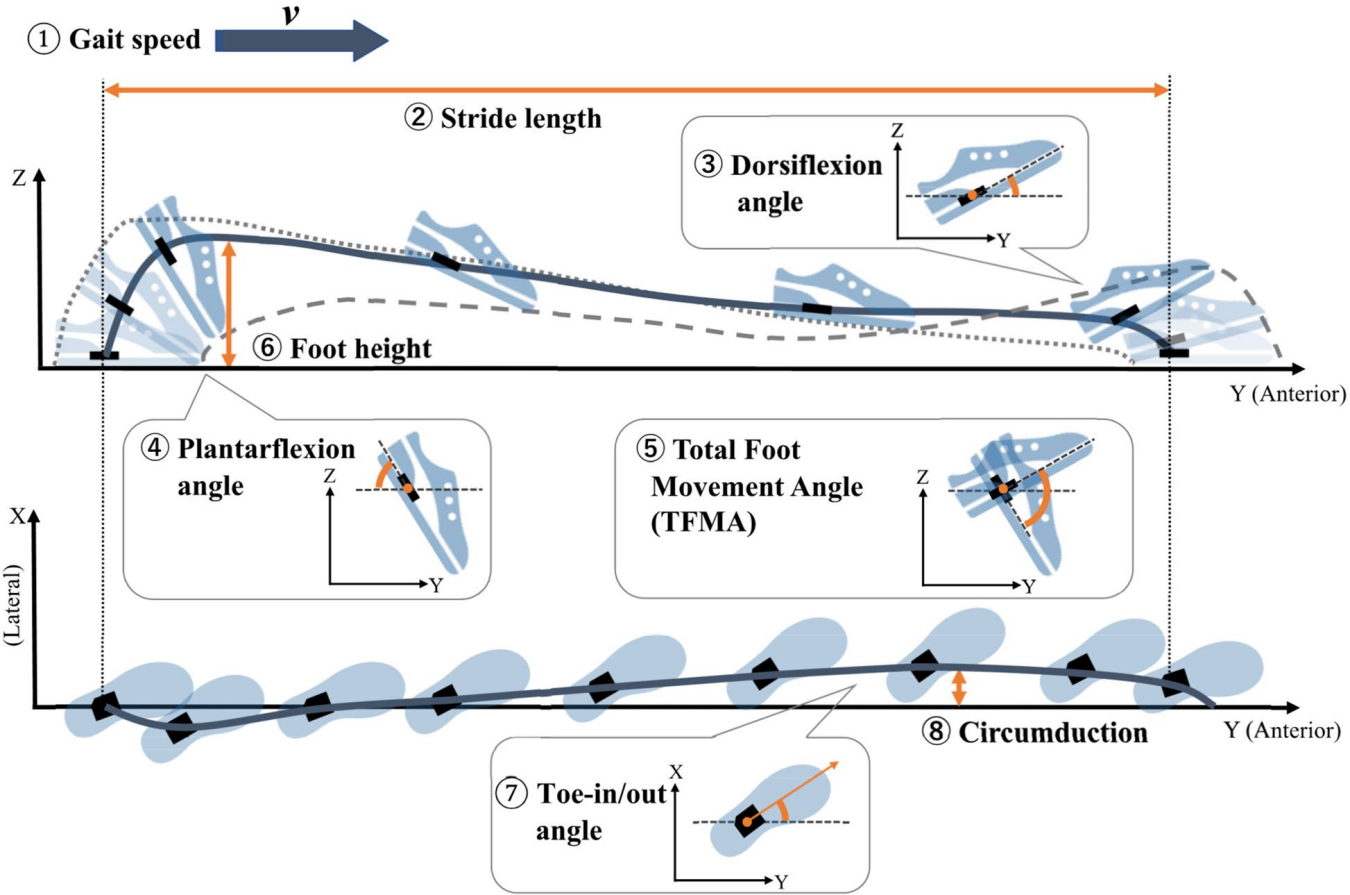
Illustration of the definition of eight gait parameters: Gait speed, stride length, dorsiflexion angle, plantarflexion angle, total foot movement angle (TFMA), foot height, toe-in/out angle, and circumduction

From the saved IMU signals, the mean of eight gait parameters of the three gait cycles was instantly calculated and stored in the smartphone, as previously described [22]. We calculated these parameters as follows (Fig. 2):

1. Gait speed: speed calculated as stride length (m) /stride time (s)
2. Stride length: distance from the starting point to the endpoint of the foot trajectory for one stride
3. Dorsiflexion angle: Peak foot angle in the dorsal direction from the ground during the swing phase
4. Plantarflexion angle: Peak foot angle in the plantar direction from the ground during the swing phase
5. Total foot movement angle (TFMA): the total of dorsiflexion and plantarflexion angle during the swing phase
6. Foot height: the maximum height of the foot trajectory
7. Toe-in/out angle: the mean angle of foot adduction/abduction in the direction of the velocity vector during the swing phase
8. Circumduction: the displacement in the medial–lateral direction during the swing phase

Besides these gait parameters, the coefficient of variance (CV; standard deviation/mean × 100) was calculated to evaluate the variability of those parameters.

### Measurement protocol

At the beginning of the assessments, all participants who visited the laboratory or hospitals completed a paper-based questionnaire regarding their general health status and falls. The questionnaire included the history of falls in the past year, frequency of stumbling, fear of falling. Falls at the time of fracture in patients with DRF were excluded from the number of falls in the past year. As for life habits, we defined regular use of tobacco and alcohol as mean consumption one or more times a week in the latest 12 months.

To check baseline functional ability and frailty, hand grip strength (HGS) [23] and Timed Up and Go (TUG) tests [24] were performed. HGS was measured in kilograms (kg) with a Jamer dynamometer (Sammons Preston, IL, USA). We assessed the HGS on the non-fractured side in the fracture group and on both sides in the control group. The mean values of the three measurements were recorded. In the TUG test, participants were allowed to practice once, and the time to complete the test was recorded twice: once at their preferred speed and once at the fastest speed. These physical tests were performed 1 month after DRF surgery in the fracture group and at the beginning of daily gait measurements in the control group.

**Fig. 3:**
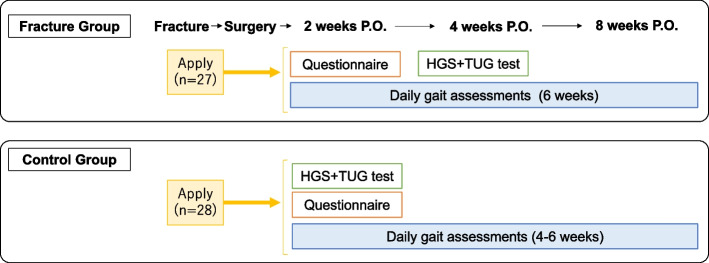
Flow diagram of the measurement protocol: The schedule of each measurement in the fracture group and in the control group. P.O. postoperative, HGS hand grip strength, TUG test Timed Up and Go test

In the daily gait assessments, we placed the IMU sensors in the dedicated insoles into both feet of their preferred shoes and provided them with a smartphone with only the original application that stored the gait data. We checked whether the participants with the sensors could walk without any problems and whether the sensors worked with the smartphone. Participants were instructed to wear their shoes with the in-shoe IMU sensors for 4 to 6 weeks as often as usual. We requested the participants to spend their daily lives as usual; therefore, we did not set the minimum time for wearing the shoes or walking with them. The measurements were carried out without the participants’ awareness, and the participants were able to see their latest gait data if they checked the smartphone. In the fracture group, daily gait assessments were started 2 weeks after DRF surgery, to allow for the effects of fractures or surgeries. Flow diagram of the measurement protocol was shown in Fig. 3.

### Data analysis

The medians of each parameter and CV in each participant were calculated. However, the automatically calculated data included those of hills, turns, and straddling, and we used Smirnov–Grubbs analysis for every gait parameter to exclude those data. After exclusion, approximately 20–1000 gait measurements were recorded per participant during the measurement period, reflecting the variations in the participants’ lifestyles. Even after the exclusion, gait data included various gait types, such as walking exercise and hurried walking; therefore, we excluded participants with less than 50 gait measurements in either foot, according to a previous report [25]. Since we computed the data from the left and right sides, participants with 100 or more gait assessments were selected for this study.

### Statistical analysis

The participants’ demographics, results of physical tests, and gait parameters were assessed using Student’s *t*-test for continuous variables in the patient’s demographics, Welch’s *t*-test for those in the gait parameters, and the chi-square test for categorical variables. Statistical significance was set at *p* < 0.05. Finally, if a significant differences between the two groups were observed, receiver operating characteristic (ROC) curves were generated to determine the optimal cutoff points for the patients with DRF according to specificity. With the identified cutoff points, sensitivity and specificity were calculated based on the Youden index. The accuracy of the ROC analysis was evaluated using areas under the curve (AUC). The odd ratio was also calculated using the cutoff value to evaluate the fracture risk.

These analyses were performed using EZR version 1.55 (Division of Hematology, Saitama Medical Center, Jichi Medical University, Japan) [26].

## Results

### Demographics and characteristics of participants

Fifty-five females participated in this study: 27 in the fracture group and 28 in the control group. There were no significant differences in age, body characteristics, and lifestyle variables between the two groups (Table 1).

**Table 1:** Patients’ demographics and characteristics

	Control	Fracture	*p*-value
	(*n* = 28)	(*n* = 27)	
Age (years) (mean, SD)	62.3 (7.0)	65.9 (8.4)	0.15^a^
Height (cm) (mean, SD)	155.2 (4.3)	156.5 (4.4)	0.28^a^
Body weight (kg) (mean, SD)	54.4 (8.0)	52.5 (8.8)	0.48^a^
Body mass index (kg/m^2^) (mean, SD)	22.6 (3.2)	21.4 (3.2)	0.23^a^
Hand dominance (right), n (%)	27 (96.4)	25 (92.6)	0.51^b^
Foot dominance (right), n (%)	23 (82.1)	25 (92.6)	0.27^b^
Smoking			
Current & previous smoker, n (%)	5 (17.9)	8 (29.6)	0.30^b^
Alcohol, n (%)	11 (39.3)	9 (33.3)	0.65^b^
Comorbidities			
Hypertension, n (%)	8 (27.6)	6 (22.2)	0.59^b^
Eye disease, n (%)	1 (3.4)	1 (3.7)	0.98^b^
Diabetes mellitus, n (%)	0 (0)	1 (3.7)	0.30^b^

*p*-values < 0.05 are considered significant

^a^Values are presented as means and standard deviations (SD). Independent Student’s *t* test was used to compare the groups

^b^Values are presented as the number of patients and percentages, and chi-squared test was used for analysis between the groups

### Fall history and functional ability

The participants in the fracture group demonstrated a significantly higher experience of falls. In the functional physical tests, HGS in the fracture group was lower than those in the control group, while there were no significant differences in the TUG test (Table 2).

**Table 2:** Fall history and physical tests

	Control	Fracture	*p*-value
	(*n* = 28)	(*n* = 27)	
The experience of fall in the past year	0 (0%)	5 (18.5%)	0.017^b^
(The number of falls)		(Once:2, twice:2, three times:1)	
The experience of stumbling	17 (60.7%)	17 (63.0%)	0.86^b^
Fear for falling	16 (57.1%)	9 (33.3%)	0.076^b^
Hand grip strength (kg) (mean, SD)	23.3 (3.4)	18.6 (4.1)	< 0.001^a^
TUG test (s) (mean, SD)			
Normal speed	8.07 (1.33)	8.08 (1.23)	0.97^a^
Faster speed	6.23 (0.89)	6.59 (1.12)	0.19^a^

*p*-values < 0.05 are considered significant

^a^Values are presented as means and standard deviations (SD). Independent Student’s *t* test was used to compare the groups

^b^Values are presented as the number of patients and percentages, and chi-squared test was used for analysis between the groups. TUG test, Timed Up and Go test

### Daily-life gait assessments

Among the participants with 100 or more gait measurements, there were no differences in the number of measurements between the two groups (Table 3). In the daily-life gait assessments, dorsiflexion (*p* = 0.014), plantarflexion angles (*p* = 0.023), and TFMA (*p* = 0.005) in the fracture group were significantly lower than those in the control group, while there were no differences in the CV.

**Table 3:** Daily-life spatiotemporal data

Variables (mean, SD)	Control	Fracture	*p*-value*
	(*n* = 28)	(*n* = 27)	
**Number of measurements**	479.3 (432.5)	601.0 (423.6)	0.31
**Mean of each parameter**			
Gait speed (m/s)	1.28 (0.12)	1.22 (0.09)	0.08
Stride length (m)	1.26 (0.12)	1.21 (0.09)	0.13
Dorsiflexion angle (degree)	25.8 (4.05)	22.7 (4.66)	0.014
Plantarflexion angle (degree)	74.8 (6.21)	71.3 (4.36)	0.023
TFMA (degree)	100.8 (8.42)	94.3 (7.76)	0.005
Foot height (cm)	14.0 (1.06)	13.4 (1.36)	0.066
Circumduction (cm)	2.82 (0.85)	3.15 (0.69)	0.13
Toe-in/out angle (degree)	13.4 (4.67)	14.4 (3.55)	0.38
**CV of each parameter (%)**			
CV Gait speed	15.2 (4.76)	15.6 (3.29)	0.70
CV Stride length	10.2 (2.84)	11.6 (2.97)	0.085
CV Dorsiflexion angle	20.8 (6.04)	23.8 (5.57)	0.063
CV Plantarflexion angle	8.52 (2.70)	10.02 (4.06)	0.12
CV TFMA	8.79 (2.54)	10.28 (2.90)	0.053
CV Foot height	8.03 (2.11)	8.86 (2.46)	0.20
CV Circumduction	51.2 (12.6)	47.2 (8.20)	0.18
CV Toe-in/out angle	30.4 (15.1)	36.8 (25.2)	0.27

*SD* Standard deviation, *TFMA* Total foot movement angle, *CV* Coefficient of variance

*p*-values < 0.05 are considered significant

^*^Independent Welch's *t* test

### Cut off values of foot angles

**Fig. 4:**
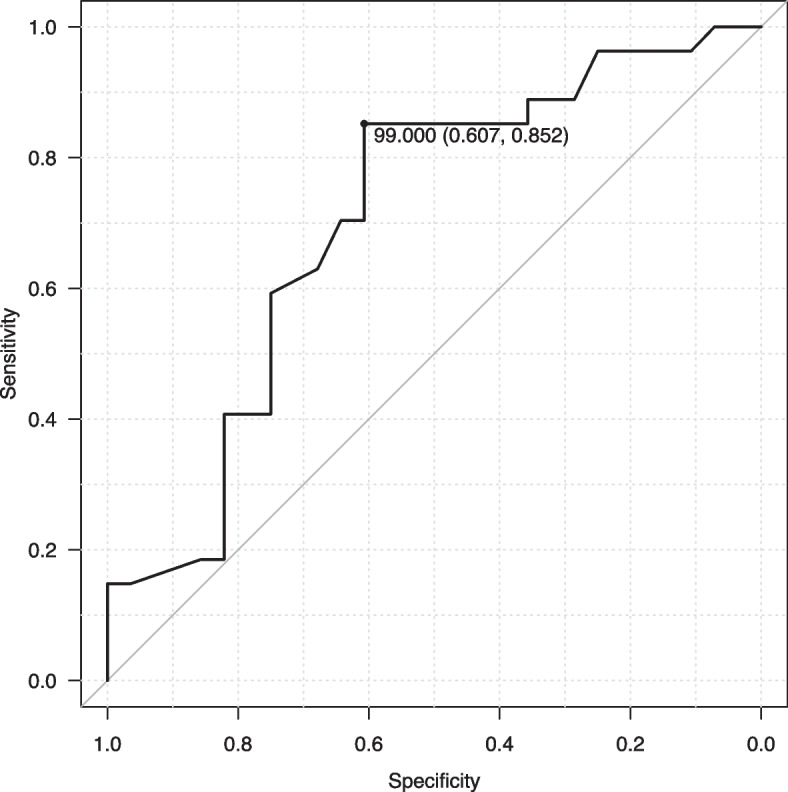
Receiver operating characteristic (ROC) curve for the total foot movement angle (TFMA). The area under the ROC curve (AUC) was 0.705 (95% confidence interval = 0.563–0.847)

According to the daily-life gait assessments, we selected TFMA for evaluating the association. ROC curve analyses of the relationship between fracture and foot angles revealed a cutoff value of fracture risk of 99.0 degrees with a sensitivity of 85.2%, specificity of 60.7%, and AUC of 0.705 (95% CI: 0.563–0.847) (Fig. 4). Using the cutoff values of 99.0 degrees of TFMA, the odds ratio of the fracture risk was 6.80 (95% CI: 1.98–23.31, *p* = 0.002).

## Discussion

We performed daily-life gait assessments using an in-shoe IMU sensor for patients with and without DRFs. Participants in the fracture group experienced more falls and had lower HGS than those in the control group, while there was no significant difference in the TUG test or other demographics. The daily-life gait characteristics in the fracture group were lower dorsiflexion and plantarflexion angles in the swing phase.

The physical characteristics of patients with DRF in this study were similar to those in previous studies [27–29]: patients with DRF demonstrated decreased HGS. The differences in HGS may suggest that the participants were representative of each group in spite of the participants’ very healthy profiles in this study. In the laser TUG test, several gait declines, such as slower speed, more steps, and asymmetric trajectory in patients with DRF, were observed [28], while no significant decline was observed in the TUG test in this study. It may be difficult to distinguish a slight decline in physical function in patients with DRF using only the time of the TUG test. Furthermore, fracture patients experienced more falls, as expected. However, approximately half of the patients with DRF experienced fractures as a first fall. Therefore, not fallers but those with DRF were effective in evaluating the precise risk related to subsequent fractures.

One of the new findings of this study was the lower dorsiflexion and plantarflexion angles during the swing phase in the fracture group. This accurate foot trajectory in the real world was revealed thanks to the in-shoe IMU sensors. These slight differences, even in healthy females, may be the very characteristics of daily-life gait in patients with wrist fractures who are at the early stage of the fracture chain. With the development of wearable sensors, studies related to gait assessments in daily life have increased in recent years. Most of these studies, however, focused only on pace and rhythm parameters, such as gait speed, stride length, and double stance phase [21, 30–33]. Generally, people transfer their foot forward with continued neutral dorsiflexion by pushing off at the initial swing phase in a normal gait [34]. The lower angle of the foot in this study may be the result of a decreased ability to control lower-limb locomotion and be the cause of stumbling. The lower angle of the foot measured by an in-shoe sensor may be effective in the risk screening for subsequent fractures.

The foot movement in the swing phase below 99.0 degrees may be related to the risk of subsequent fractures, according to our results in this study. Since we considered the foot movement during daily-life gait as a series of movements rather than strictly divided into the dorsiflexion and plantarflexion angles, we selected the total foot movement for the evaluation of the cutoff values of foot angles in this study. However, we measured the angle of the IMU sensors in the insoles, which were the combined results of the hip, knee, ankle, and other joints. The TFMA of healthy people was over 100 degrees in general [22], although few previous studies focused on the angles of insoles. TFMA in the swing phase may be useful as an indicator of the subsequent fracture. Further research is needed for the risk assessment tool for fractures using the in-shoe IMU sensor outside the hospital.

As for the CVs of each gait parameter, there were no significant differences in this study, while previous reports with laboratory gait demonstrated an increased CV of more parameters, such as stride length and double stance phase, related to fall risk [14, 35]. The differences between previous reports and our findings may be due to various factors that affect gait in daily life. The obtained data in this study include various factors, such as environmental and psychiatric factors, which may mask the true differences in the variability of the participant’s gait. Which factors and how they affect daily-life gait should be further explored to predict the risk of subsequent fracture more precisely.

This study had some limitations. First, this study had a small number of very healthy participants and only females, which may limit generalizability to other aging populations. However, females are one of the risk factors for falling among community-living older adults [36]. The findings in this study may be the first step toward future longitudinal and interventional studies. Second, we evaluated the daily-life gait by only eight parameters and CVs. It is not clear which or what parameters would be useful for the evaluation of fracture risk or how the parameters interact with each other. We would like to establish further research methodology to explore gait parameters in daily life that can reveal risk factors for subsequent fractures.

## Conclusions

In this study, we performed a case–control study on daily-life gait analysis in postmenopausal females with and without fragility fractures. Owing to the in-shoe IMU sensor, we could extract foot kinematics in daily-life gait without participants’ awareness. Patients with fractures demonstrated a lower angle of dorsiflexion and plantarflexion in the swing phase, which may cause fragility fractures. In addition, we revealed that lower TFMA, under 99.0 degrees, was the risk of subsequent fracture. These slight differences, which could not be identified merely by observation, may lead to future injurious falls. Therefore, the TFMA may be a good parameter in the fracture risk assessment. Further studies to predict and prevent subsequent falls or fractures before they occur from daily-life gait are warranted.

## Data Availability

All data generated or analyzed during this study are available from the corresponding author upon request, if legally and ethically possible. Each author warrants that this work is original. Neither this work nor a similar work by the authors has been published elsewhere in any language nor shall be submitted for publication elsewhere while under consideration by BMC Musculoskeletal Disorders.

## Abbreviations

DRF: Distal radius fracture
IMU: Inertial measurement units
TFMA: Total foot movement angle
HGS: Hand grip strength
TUG test: Timed Up and Go test
